# Pharmacokinetics and Safety of Rifamycin SV after Single and Multiple Doses of MMX^®^ Modified Release Tablets in Healthy Male and Female Volunteers

**DOI:** 10.3390/antibiotics10020167

**Published:** 2021-02-06

**Authors:** Andrea Francesco Daniele Di Stefano, Milko Massimiliano Radicioni, Angelo Vaccani, Alessandro Mazzetti, Luigi Maria Longo, Luigi Moro

**Affiliations:** 1 CROSS Research S.A., Via F. A. Giorgioli, 14, CH-6864 Arzo, Switzerland; milko.radicioni@croalliance.com (M.M.R.); angelo.vaccani@croalliance.com (A.V.); 2 Cosmo Technologies Ltd., Riverside II, Sir John Rogerson’s Quay, Dublin 2, Ireland; amazzetti@cassiopea.com (A.M.); llongo@cosmopharma.com (L.M.L.); lmoro@cosmopharma.com (L.M.)

**Keywords:** non absorbable antibiotics, rifamycin SV, pharmacokinetics, healthy subjects, MMX

## Abstract

The primary objective of this single- and multiple-dose pharmacokinetic study was the investigation of rifamycin SV’s pharmacokinetic profile in plasma and urine. All the 18 enrolled healthy men and post-menopausal women received modified release tablets containing 600 mg of the oral non-absorbable antibiotic, rifamycin SV, according to a multiple dose regimen: one tablet three times a day (daily intake: 1800 mg) for 14 consecutive days. Blood sampling and urine collection were performed up to 24 h post-dose after the first dose on Days 1 and 7. On average, on Day 1, C_max,0–24_ was 5.79 ± 4.24 ng/mL and was attained in a median time of 9 h. On Day 7, all the subjects had quantifiable levels of rifamycin SV in plasma at each sampling time. After a peak concentration attained 2 h post-dose (mean ± SD concentration: 10.94 ± 16.41 ng/mL), rifamycin SV decreased in plasma to levels similar to those of Day 1. The amounts of rifamycin SV excreted in urine paralleled the plasma concentration at the corresponding times. On Day 1, the total amount excreted in urine was 0.0013%, and was 0.0029% on Day 7. The study results confirmed those of the previous Phase I study: the systemic absorption of rifamycin SV was also proved negligible after 7 days of the 600 mg t.i.d. dose regimen of the newly formulated tablets, currently under development for the treatment of several small and large intestinal pathologies, including diarrhea-predominant irritable bowel syndrome, hepatic encephalopathy, and others. Registered at ClinicalTrials.gov with the identifier NCT02969252, last updated on 26JAN18.

## 1. Introduction

Rifamycin SV, used as sodium salt, is a semisynthetic antibiotic belonging to the class of ansamycins obtained from rifamycin B, which is produced in the fermentation broth of *Streptomyces mediterranei* (or also *Amycolatopsis Mediterranei* or *Nocardia Mediterranea* n. sp.) [1,2]. Rifamycin SV, endowed with a broad-spectrum activity against Gram-positive and Gram-negative bacteria and mycobacteria, was introduced into clinical practice in 1962 as an anti-infective drug.

The activity of rifamycin SV is predominantly bactericidal because of its interference with bacterial protein synthesis: the rifamycin family, like other ansamycins, inhibit protein synthesis by binding the β subunit of bacterial DNA-dependent RNA polymerase.

Antimicrobial drugs represent an important treatment approach and remain the current standard of care for some types of enteric infection [3], including shigellosis, diarrhea due to infection with enterotoxigenic *Escherichia coli*, cholera, antibiotic-associated colitis due to *Clostridium difficile* proliferation, traveler’s diarrhea, giardiasis, and amoebiasis [3]. Since the early 1980s, non-absorbable antibiotics have represented an effective treatment of infectious acute diarrhea [3,4] inasmuch as they limit the antimicrobial effect to the intestinal lumen, avoiding systemic effects. Thanks to a multi-matricial core composition, surrounded by a pH-sensitive coating, MMX tablets arrive unaltered to the terminal part of the ileum, where they start to gradually release rifamycin SV, which continues during the tablet’s transit from the terminal ileum onward. Thus, this specific formulation, designed to deliver the non-absorbable antibiotic directly into the lumen of the large intestine districts, as previously demonstrated by pharmaco-scintigraphic studies of other active substances [5,6], offers consistent advantages over other formulations, and thus minimizing the side effects closely related to unwanted activity against the saprophytic flora living in the upper intestinal tract, and improving the drug’s efficacy through an intestine-targeted topical delivery.

The efficacy and safety of sodium rifamycin 200 mg tablets administered four times a day (q.i.d.) for 3 days to patients with infectious diarrhea were previously investigated in comparison with rifaximin tablets administered according to the same dose regimen [7].

In addition, the pharmacokinetics of rifamycin SV was previously investigated in healthy male and female subjects after single and multiple administrations of the 200 mg tablets [8]. Oral rifamycin SV was confirmed to have a negligible oral systemic bioavailability, further reduced by the MMX formulation that focuses the drug release in the colon lumen. At the end of 2018, rifamycin SV MMX^®^ 200 mg modified release tablets were approved in the USA and several European countries for the treatment of patients with travelers’ diarrhea caused by non-invasive strains of *Escherichia coli*.

The present trial aimed at investigating the pharmacokinetics of the antibiotic after single and multiple doses, three times a day (t.i.d.) for 14 days, in healthy volunteers taking the newly formulated 600 mg MMX^®^ modified release tablets, which contain a higher amount of sodium rifamycin SV. The test formulation differed from the recently approved 200 mg tablets, used in the previous studies [7,8], not only in the antibiotic strength, but also in the core matrix composition, that allows the active ingredient progressive release to start at pH 6 instead of 7. This change allows a drug release starting in the small intestine, i.e., in a more proximal enteric region than with the former tablets, which may improve the biological effect of rifamycin SV on pathogen foci also infecting the small intestine.

The newly formulated tablets are currently under development for the treatment of diarrhea-predominant irritable bowel syndrome (IBS-D) and other intestinal pathologies with an infective component. Indeed, an increasing amount of data suggests that IBS is commonly associated with small intestinal bacterial overgrowth (SIBO), and that a bacterial overgrowth may be part of IBS pathogenesis [9,10,11]. Under normal conditions, the bacterial concentration in the small intestine is not higher than 10^2–3^ colony forming units (CFU) per mL [12]. A SIBO is defined as a finding of coliform bacteria ≥1 × 10^3^ CFU/mL of proximal jejunal aspirate [9]. Bacteria are allegedly involved in the pathogenesis of IBS through the metabolic capacity of the luminal microbiota and the potential of the mucosa-associated microbiota to influence the host via immune–microbial interactions [13,14]. Indeed, the prevalence of SIBO in IBS varies from 30% to 85%, according to the source [9,15,16,17,18]. The possibility to administer rifamycin SV in a new oral formulation designed to deliver the non-absorbable antibiotic directly into more intestinal districts, including the terminal part of the small intestine, is expected to offer consistent advantages over the formulations currently used to treat IBS, and to improve the drug efficacy due to direct topical delivery, without exposing the patients to unwanted systemic effects thanks to the negligible drug absorption.

## 2. Methods

### 2.1. Study Design

The present study was a single and multiple dose, open-label, pharmacokinetics and safety Phase I study. The primary objective was the investigation of the kinetic profile of rifamycin SV in plasma and urine, and the safety and tolerability of the new 600 mg MMX^®^ tablets upon oral administration. The study included a screening visit, a confinement of 7 days and 8 nights, and 2 ambulatory visits for a minimum study duration of 17 days. All the study subjects received rifamycin SV 600 mg tablets according to the following multiple dose regimen: one tablet 3 times a day (t.i.d., for a total daily intake of 1800 mg) for 14 consecutive days, starting from Day 1. The blood sampling and the urine collection schedule were planned up to 24 h post-dose after the 1st dose on Days 1 and 7, considering the previously published kinetic data [8], and aiming to describe the antibiotic kinetic profile at the steady state and assuming its attainment within 7 days with this dose schedule. On Day 8, after the multiple-dose blood and urine sampling for pharmacokinetic measurements, the subjects left the Phase I Unit, and then returned for 2 ambulatory visits scheduled on Days 11 and 15. On Day 15, the subjects underwent the final study visit. The treatment continued for further 7 days with the aim of collecting safety measures of the treatment.

According to previous experience, the safety assessments of the present study were focused on the recording of adverse events, on clinical laboratory assays, and on the measurement of vital signs.

#### 2.1.1. Study Population and Criteria for Inclusion

The study was performed at the Phase I Unit of CROSS Research S.A., Arzo, Switzerland.

Healthy men and post-menopausal women were included into the trial according to the following main inclusion criteria: (i) age of 18 to 55 y; (ii) a body mass index inside the range 18.5–30 kg/m^2^; (iii) good health based on medical history, physical examination, a 12-lead electrocardiogram (ECG), and routine hematology and blood chemistry tests; (iv) willingness to provide written informed consent.

Main exclusion criteria were the standard criteria for bioavailability estimation of new drugs, namely: (i) intake of any medication; (ii) a history of drug, caffeine (>5 cups of coffee or tea/day) or tobacco (≥10 cigarettes/day) abuse; (iii) history of alcohol consumption in excess of two drinks per day for men, and of one drink per day for women, as defined by the U.S.D.A. dietary guidelines [19].

The study was descriptive and non-comparative. Therefore, the sample size was not derived from a statistical power calculation. A total of 18 subjects were planned to be included in the study.

The planned population included healthy men, and healthy sterile or post-menopausal women. The exclusion of fertile women was aimed at preventing potential drug–drug interactions of rifamycin SV with oral hormonal contraceptives as rifamycin SV, like the other ansamycins, is a metabolic inducer which could affect the bioavailability of other compounds.

#### 2.1.2. Investigational Treatments and Dose Regimen

All the study subjects took 1800 mg of sodium rifamycin SV as one 600 mg tablet t.i.d. for 14 consecutive days at 08:00 ± 1 h, 14:00 ± 1 h, and 20:00 ± 1 h, starting from Day 1. Each dose was administered with half a glass (≈150 mL) of still mineral water.

The administrations from Day 1 to the morning of Day 8 were performed by the investigator or deputies at the Phase I Unit.

After discharge on Day 8, the subjects continued the treatment according to the same dose regimen at home up to Day 14, for a total of 42 administrations.

The planned dose regimen foresaw a daily intake of 1800 mg of sodium rifamycin SV, which is similar to the maximal dose recommended for various injectable preparations of sodium rifamycin available in Europe. The planned intervals between the 3 daily doses (6 h + 6 h + 12 h) aimed at standardizing the dosing conditions throughout each treatment day. In fact, maintaining a τ of 6 h between the first and the second dose, and also between the second and the third, allowed for each dose to be administered in fasting conditions before each meal. During the confinement, standardized meals were always served after each dosing.

### 2.2. Ethical Procedures

The study CRO-PK-15-294–Sponsor code CB-01-11/27 was approved by the independent ethics committee of Canton Ticino on 24 February 2015. Ref. nr. 2888. The Swiss Federal Health Authorities (Swissmedic) authorized the study on 25 June 2015 and assigned the reference number 2015DR1084. The study was conducted in compliance with the Swiss ordinance on clinical trials in human research, and in accordance with the Declaration of Helsinki and the general principles of the ICH Harmonised Tripartite Guidelines for Good Clinical Practice (GCP). Subjects did not undergo any study procedure before signing the written informed consent form. The first subject was enrolled on 9 July 2015 and the last subject completed the trial on 17August 2015.

### 2.3. Sample Collection, Handling, and Analytics 

Plasma concentrations of rifamycin SV were measured at: pre-dose (0), 1, 2, 3, 4, 6, 7, 8, 9, 10, 12, 13, 14, 15, 16, 18, and 24 h post-dose after the first dose on Days 1 and 7.

Blood sampling at 7, 8, 9, 10 and 12 h post-dose followed the second dose on Days 1 and 7. Blood sampling at 13, 14, 15, 16, 18, and 24 h post-dose followed the third dose on Days 1 and 7.

Urine concentrations of rifamycin SV were measured during the following time intervals: 0–3, 3–6, 6–12, 12–18, and 18–24 h post-dose after the first dose on Days 1 and 7.

Blood samples were collected using an indwelling catheter with a switch valve into K2-EDTA tubes, and stored on ice for a maximum of 20 min. Then, the samples were centrifuged at 4 °C for 10 min at 2500 g to obtain plasma. Each plasma sample was immediately divided into 3 aliquots in pre-labelled amber polypropylene tubes, and stored frozen at ≤ −70 °C pending analyses.

During each interval, urine was collected into containers, and kept refrigerated. At the end of each collection interval, urine volume was measured, 1% of a 1% Tween-20 solution was added to prevent rifamycin SV absorption in the container material and, after thorough mixing, 3 aliquots were prepared in amber polypropylene tubes. The aliquots were stored at ≤ −70 °C until analyses.

The concentration of rifamycin SV in plasma and urine was determined at the ABL Analytisch Biochemisch Laboratorium, the Netherlands, by a validated LC–MS/MS method, also used in the previous study [8]. The lower quantification limit for the determination in plasma and urine (0.5 ng/mL) was far lower than in the previous study.

### 2.4. Pharmacokinetics Variables and Data Analysis

The following pharmacokinetic parameters (Table 1, Table 2 and Table 3) were measured and/or calculated for plasma rifamycin SV after the first dose, according to a non-compartmental model, and using the validated software WinNonLin^®^ 6.3 (Pharsight Corporation):

The following parameters were measured and/or calculated for plasma rifamycin SV after the 3 multiple doses of Day 7.

The following parameters were measured/calculated for urine rifamycin SV after the first 3 doses (Day 1), and after the 3 multiple doses of Day 7.

### 2.5. Safety Variables

The safety variables included: the recording of adverse events during the whole study duration; blood pressure, heart rate, and body weight measurements at screening, pre-dose and upon discharge; physical examinations; ECG; and routine blood chemistry, hematology, and urinalysis laboratory assays performed at screening, and upon the end of the study.

## 3. Results

### 3.1. Disposition of Subjects

Eighteen (18) Caucasians, aged 22 to 55 y were enrolled as planned. Baseline demographic data are summarized in Table 4.

All 18 subjects received the planned treatment and completed the study according to the protocol.

### 3.2. Rifamycin SV Plasma Kinetic Profile

**Figure 1:**
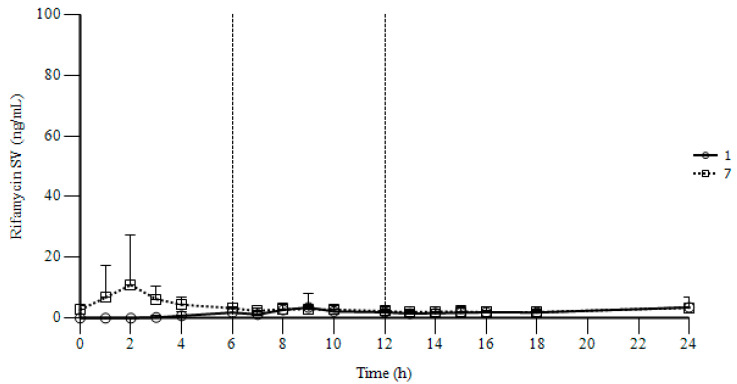
Mean (+SD) rifamycin SV concentration (ng/mL) vs. time profiles on Day 1 (○ solid line) and Day 7 (□ dashed line) of treatment with 600 mg modified release tablets t.i.d. (times a day). Linear scale. N = 18; 1: rifamycin SV concentrations on Day 1; 7: rifamycin SV concentrations on Day 7; Dashed vertical lines indicate the timing of the second and the third daily doses.

Mean rifamycin SV concentration (ng/mL) vs. time profiles on Days 1 and 7 of treatment are shown in Figure 1 in linear scale. The main parameters of plasma rifamycin SV on Days 1 and 7 of the treatment are presented in Table 5.

The plasma rifamycin SV concentrations vs. time curves could not be extrapolated to infinity for any subject at the established conditions.

On Day 1, quantifiable levels of rifamycin SV were first found in plasma 3 h after the first dose. The plasma concentration of the antibiotic increased slowly over the second dose and attained a peak 9 h post-dose (3.51 ± 4.55 ng/mL), i.e., 3 h after the second dose. After the sampling at 9 h post-dose, rifamycin SV decreased slowly in plasma over the third dose.

On Day 7, as expected, all the subjects had quantifiable rifamycin SV concentrations at pre-dose (2.73 ± 1.54 ng/mL). After the morning dose, rifamycin SV concentration increased rapidly in plasma, and attained a first peak concentration 2 h post-dose (10.94 ± 16.41 ng/mL). At 2 h post-dose, one third of the subjects had plasma rifamycin SV concentrations >10 ng/mL, which in one case reached a concentration >70 ng/mL. Afterwards, the antibiotic decreased in plasma, and from 7 to 18 h post-dose ranged between 1.90 and 2.98 ng/mL. Pre-dose and 24 h post-dose concentrations showed that systemic rifamycin SV was at the steady state.

When rifamycin SV parameters of Day 1 (interval 0–6 h post-dose) are compared with those of Day 7 (interval 12–18 h post-dose), a slight increase in the exposure to rifamycin SV can be observed after 7 days of treatment. In detail, the rate of absorption of the analyte (C_max,0–6_ of Day 1 vs. C_max,12–18_ of Day 7) increased by 1.32 times, and the extent of absorption (AUC_0–6_ of Day 1 vs. AUC_ss,12–18_ of Day 7) increased by 3.73 times. These data show that the rifamycin SV accumulation after 7 days of t.i.d. treatment was very limited. When AUC_0–24_ of Day 1 is compared with AUC_ss,0–24_ of Day 7 an increase in extent of absorption of rifamycin SV by 1.83 can be observed. This increase in rifamycin SV AUC from Day 1 to Day 7 is partly due to the notable concentration peak at 2 h post-dose on Day 7, mentioned above. Otherwise, the curve of plasma concentration of rifamycin SV vs. time on Day 7 is almost superimposable on that of Day 1 from 8 h to 24 h post-dose.

### 3.3. Rifamycin SV Urinary Excretion

**Figure 2:**
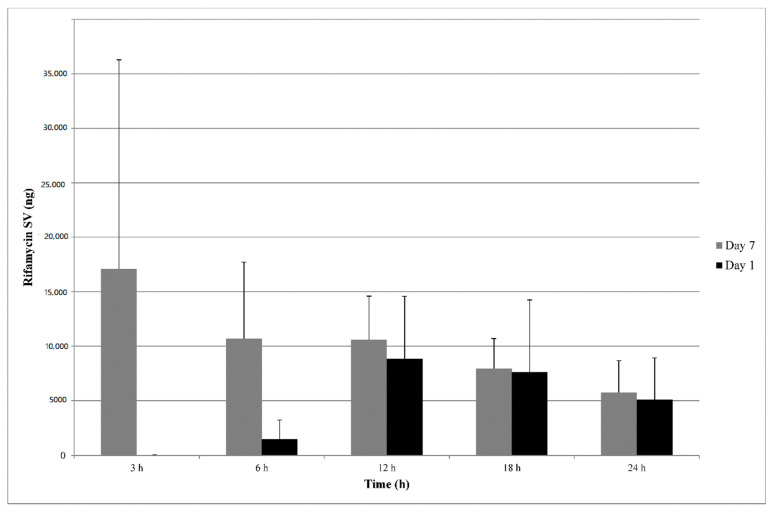
Mean (+SD) rifamycin SV excretion in urine (ng) vs. time profiles on Day 1 (black) and Day 7 (grey) of treatment. N = 18; Day 1: rifamycin SV concentrations on Day 1; Day 7: rifamycin SV concentrations on Day 7.

Mean rifamycin SV excreted amounts on Days 1 and 7 are shown in Figure 2. Main parameters of urine rifamycin SV on Days 1 and 7 are presented in Table 6.

The excretion of rifamycin SV on Day 1 attained a peak in the 6–12 h post-dose collection interval, i.e., after the second dose of Day 1, with an amount of 8838.17 ± 5727.87 ng found in urine. The peak observed in the 6–12 h interval corresponded to the peak observed in plasma concentrations. Afterwards, the excretion rate was constant and small in amount.

On Day 7, the excretion of rifamycin SV showed a maximum in the first collection interval (between 0–3 h post-dose with a mean amount of 17,100.56 ± 19,169.28 ng). The peak observed in the 0–3 h interval is consistent with the peak observed in plasma concentrations at 2 h post-dose. Afterwards, excretion decreased in the 3–6 h interval and after 12 h had levels similar to those observed on Day 1 in the collections between 6 and 24 h post-dose. Between 6 and 24 h post-dose, the rate of rifamycin SV urinary excretion was on average constant.

The total amount of rifamycin SV excreted on Day 7 of the treatment increased on average by 2.3 times, compared to the amount excreted on Day 1, which is consistent with the plasma concentration data.

### 3.4. Safety

Adverse events occurred to 6/18 subjects (33.3%). The majority of reported adverse events were not judged to be related to the treatment.

The reported adverse events are summarized by system organ class and preferred term in Table 7.

The most common event was headache. All the three reported episodes started within about 5 h of the previous treatment dose. One of the headache episodes was of moderate intensity, while the other two were mild and resolved spontaneously after about 12 h. None of the headache episodes was judged as related to the treatment.

During the study treatment, some abnormalities in the measured clinical laboratory assays were judged as non-clinically significant by the investigator, with the exception of five urine parameters for one subject who suffered from cystitis on the last treatment day. In detail, the presence of hematic pigments and leukocytes in urine, and of leukocytes, erythrocytes, and bacteria in the urinary sediment found at the final visit analysis was abnormal and clinically significant. However, all five abnormalities were signs of the current cystitis with stranguria and were unrelated to the study treatment.

No notable change in any other laboratory parameter was observed between the final laboratory analysis and the screening. As expected with non-absorbable drugs, no significant effect of rifamycin SV on any laboratory parameter was observed during the study.

No significant change in vital signs, body weight, or ECG was observed after treatment with rifamycin SV for 14 consecutive days.

## 4. Discussion

The new dosage form of rifamycin SV was administered for 14 consecutive days for the first time in the present study, which also took advantage of a more sensitive LC–MS/MS analytical method for the assay of the antibiotic in plasma and urine.

Healthy post-menopausal women and men enrolled in the present study were exposed to a daily dose of 1800 mg of rifamycin SV for 14 consecutive days, thus allowing the investigation of the kinetic profile of the systemically available rifamycin SV, both after the first dose, and after 7 days of treatment, and the collection of extended safety information after further 7 days of treatment with the new formulation.

The plasma concentrations of rifamycin SV observed in the present study were consistent with the results of the previous Phase I study [8] with a different formulation, published in 2011, and confirmed that the systemic absorption after oral administration of rifamycin SV is also negligible after 7 days of a t.i.d. treatment with the new rifamycin SV tablets for a daily dose of 1800 mg. Being a poorly absorbed drug, the concentration values reached by rifamycin SV in plasma were relatively low after the first dose on Day 1 of the treatment. The highest mean concentration of rifamycin SV measured in plasma on Day 7 was 10.94 ± 16.41 ng/mL (2 h post-dose), while the highest individual concentration of rifamycin SV measured in plasma was 72.60 ng/mL (2 h post-dose). Remarkably, the highest mean concentration (10.94 ± 16.41 ng/mL) was approximately 3000 times lower than C_0_ (36,025.00 ± 8569.32 ng/mL) measured 5 min after a single i.v. 250 mg dose of the antibiotic [8]. The mean rifamycin SV concentration value of the present study was 10.94 ± 16.41 ng/mL, which is on average as low as the mean concentration of 12.55 ± 3.66 ng/mL, which was measured 8 h after a single i.v. dose of 250 mg of the antibiotic in the previous study. Finally, the systemic exposure to rifamycin SV measured in the present study confirmed the negligible systemic absorption of the antibiotic, as proved by both the plasma concentrations and the urinary elimination data obtained at steady state. In fact, the mean percentage amount of rifamycin SV excreted in urine was 0.0013% of the administered dose on Day 1 and increased to 0.0029% on Day 7. The amount of rifamycin SV excreted in urine after 7 days of the t.i.d. treatment with a daily dose of 1800 mg was on average 52,036 ± 22,631 ng, i.e., 0.0029 ± 0.0013% of the dose. The same parameter obtained in the previous Phase I study after a single i.v. dose of rifamycin SV (250 mg) was on average 2.62 ± 0.86% and ranging between 1.0176 and 4.9129% [8]. The comparison between the excreted fractions of the two studies confirms that, after 7 days of a t.i.d. treatment, the percentage of systemically absorbed rifamycin SV with a 1800 mg daily dose is also negligible.

Notably, even the tablet formulation, renewed in the core matrix composition, that allowed the active ingredient progressive release to start at pH 6 instead of 7, i.e., in the ileum, did not have any significant impact on the peculiar rifamycin kinetics, inasmuch as this change did not enhance the antibiotic systemic absorption.

Safety data of the present trial were also in agreement with the results of the previous Phase I multiple dose study [8], in fact considering that a higher daily dose (1800 mg vs. 800 mg) was administered in the present study and for a longer time (14 days vs. 4 days), the number and severity of adverse events were confirmed as low, mild, and short lasting. In fact, considering the adverse event most frequently reported in the present study and in the previous one, headache had a frequency ranging 4.2–8.3% in the latter, and of 16.7% in the former.

The results of the present study are in accordance with those of the previous studies, thus confirming an excellent safety profile. Furthermore, no severe adverse events were reported, and no subject discontinued the study due to safety concerns. Some abnormal urine parameters found after treatment were signs of an episode of cystitis, which was unrelated to the treatment. No significant change in any other laboratory parameter was observed. No significant change in vital signs, body weight, or ECG was observed after treatment. No significant change in liver or kidney functions was observed during the 14th day treatment.

In conclusion, as evidenced in particular by the plots, which are unable to show any characteristic curves due to the reduced amount of the antibiotic in the systemic circulation, and by the pharmacokinetic parameters, the study results showed that the absorption of rifamycin SV was very low, and also after a multiple-dose treatment. With respect to the kinetics of any other drug claiming systemic efficacy, the feature of negligible systemic absorption is generally regarded as an inadequacy or a failure. On the contrary, it is indeed to be considered as beneficial and supportive in the case of rifamycin SV, which is expected to display high local efficacy and low systemic unwanted effects. The negligible rifamycin SV absorption shown herewith after a t.i.d. multiple-dose treatment for 14 days, makes the antibiotic also suitable for additional gastroenteric indications, such as several small and large intestinal pathologies, including diarrhea-predominant irritable bowel syndrome, hepatic encephalopathy, and others.

## Figures and Tables

**Table 1:** Pharmacokinetic parameters planned to be measured and/or calculated for plasma rifamycin SV after single dose.

C_max,0–6_:	Maximum plasma concentration achieved in the selected interval (0–6 h) after first dose
C_max,0–24_:	Maximum plasma concentration achieved in the selected interval (0–24 h) after first, second, and third doses
t_max,0–6_:	Time to achieve C_max,0–6_
t_max,0–24_:	Time to achieve C_max,0–24_
AUC_0–6_:	Area under the concentration/time curve during the selected interval (0–6 h) calculated with trapezoidal method
AUC_0–24_:	Area under the concentration/time curve during the selected interval (0–24 h) calculated with trapezoidal method

**Table 2:** Pharmacokinetic parameters planned to be measured and/or calculated for plasma rifamycin SV after multiple doses.

C_max,ss,12–18_:	Maximum plasma concentration in the selected interval (12–18 h) achieved at steady state: i.e., in the interval 0–6 h after the third dose administered on Day 7
C_min,ss,12–18:_	Minimum plasma concentration in the selected interval (12–18 h) achieved at steady state
AUC_ss,12–18_:	Area under the concentration/time curve during the selected interval (12–18 h) at steady state calculated with trapezoidal method
AUC_ss,0–24_:	Area under the concentration/time curve during the selected interval (0–24 h) at steady state calculated with trapezoidal method
t_max,ss,12–18_:	Time to achieve C_max,ss_,_12–18_
C_ave,12–18_:	Average plasma concentration in the selected interval (12–18 h), calculated as AUC_ss,12–18_ / τ, where τ is the dosing interval (6 h)
PTF%_12–18_:	Peak–trough fluctuation, calculated in the selected interval (12–18 h) as $\frac{C_{\max,\;ss12–18}\;-\;C_{\min,ss12–18}}{C_{\mathrm{ave},12–18}}\;\times \;100$

**Table 3:** Pharmacokinetic parameters planned to be measured and/or calculated for urine rifamycin SV after single and multiple doses.

Ae_0–24_:	Total amount of analyte excreted in urine during the selected interval (24 h)
%Ae_0–24_:	Percentage amount of analyte excreted in urine, calculated as (Ae_0–24_ / dose × 3) × 100

**Table 4:** Mean (±SD) baseline demographic data (N = 18).

Age	Height	BW	BMI	Sex	Race
(y)	(cm)	(kg)	(kg/m^2^)		
40.6 ± 11.2	171.5 ± 8.8	72.08 ± 12.18	24.42 ± 3.07	Women 4 (22.2%) Men 14 (77.8%)	White 18 (100%)

BW: body weight; BMI: body mass index.

**Table 5:** Pharmacokinetic parameters of plasma rifamycin SV measured and calculated on Days 1 and 7 of treatment (N = 18).

Parameter	Unit	Day 1	Parameter	Unit	Day 7
C_max,0–6_	ng/mL	2.19 ± 1.94	C_max,ss12–18_	ng/mL	2.90 ± 1.73
t_max,0–6_	h	6.00 (4.00–6.00)	t_max,ss12–18_	h	12.00 (12.00–18.00)
AUC_0–6_	ng/mLxh	3.26 ± 2.85	C_min,ss12–18_	ng/mL	1.55 ± 0.39
C_max,0–24_	ng/mL	5.79 ± 4.24	C_average,12–18_	ng/mL	2.02 ± 0.66
t_max,0–24_	h	9.00 (4.00–24.00)	AUC_ss,12–18_	ng/mLxh	12.15 ± 3.95
AUC_0–24_	ng/mLxh	43.67 ± 20.15	PTF%_12–18_	%	59.70 ± 42.48
			AUC_ss,0–24_	ng/mLxh	80.08 ± 34.09

**Table 6:** Total amount of rifamycin SV excreted in urine calculated on Days 1 and 7 of the treatment; mean ± SD is reported. N = 18.

Parameter	Unit	Day 1	Parameter	Unit	Day 7
Ae_0–24_	ng	23,076 ± 14,328	Ae_0–24_	ng	52,036 ± 22,631
%Ae_0–24_	% of dose	0.0013 ± 0.0008	%Ae_0–24_	% of dose	0.0029 ± 0.0013

Ae: Total amount of analyte excreted in urine.

**Table 7:** Display of adverse events. Number of subjects reporting and number of reported adverse events by system organ class and preferred term.

MedDRA* Description	Overall N = 18	
System Organ Class	Number of Adverse Events n	Subjects with Adverse Events n (%)
Preferred term		
All adverse events	9	6 (33.3)
Nervous system disorders	3	3 (16.7)
Headache	3	3 (16.7)
Gastrointestinal disorders	3	2 (11.1)
Toothache	2	2 (11.1)
Constipation	1	1 (5.6)
Musculoskeletal and connective tissue disorders	2	2 (11.1)
Back pain	2	2 (11.1)
Infections and infestations	1	1 (5.6)
Cystitis	1	1 (5.6)

*: MedDRA version 18.1.

## Data Availability

The datasets generated and analysed during the current study are not publicly available. These data are protected by a confidentiality agreement with the study sponsor, Cosmo Technologies Ltd., Ireland, due to their ethically and commercially sensitive nature. Further information about the data and conditions for access are available at www.cosmopharma.com.
